# A Low Complexity Near-Optimal Iterative Linear Detector for Massive MIMO in Realistic Radio Channels of 5G Communication Systems

**DOI:** 10.3390/e22040388

**Published:** 2020-03-28

**Authors:** Mahmoud A. Albreem, Mohammed H. Alsharif, Sunghwan Kim

**Affiliations:** 1 Department of Electronics and Communications Engineering, A’Sharqiyah University, Ibra 400, Oman; mahmoud.albreem@asu.edu.om; 2 Department of Electrical Engineering, College of Electronics and Information Engineering, Sejong University, 209 Neugdong-ro, Gwangjin-gu, Seoul 05006, Korea; malsharif@sejong.ac.kr; 3 School of Electrical Engineering, University of Ulsan, Ulsan 44610, Korea

**Keywords:** 5G, massive MIMO, detection, iterative matrix inversion methods, QuaDRiGa

## Abstract

Massive multiple-input multiple-output (M-MIMO) is a substantial pillar in fifth generation (5G) mobile communication systems. Although the maximum likelihood (ML) detector attains the optimum performance, it has an exponential complexity. Linear detectors are one of the substitutions and they are comparatively simple to implement. Unfortunately, they sustain a considerable performance loss in high loaded systems. They also include a matrix inversion which is not hardware-friendly. In addition, if the channel matrix is singular or nearly singular, the system will be classified as an ill-conditioned and hence, the signal cannot be equalized. To defeat the inherent noise enhancement, iterative matrix inversion methods are used in the detectors’ design where approximate matrix inversion is replacing the exact computation. In this paper, we study a linear detector based on iterative matrix inversion methods in realistic radio channels called QUAsi Deterministic RadIo channel GenerAtor (QuaDRiGa) package. Numerical results illustrate that the conjugate-gradient (CG) method is numerically robust and obtains the best performance with lowest number of multiplications. In the QuaDRiGA environment, iterative methods crave large n to obtain a pleasurable performance. This paper also shows that when the ratio between the user antennas and base station (BS) antennas (β) is close to 1, iterative matrix inversion methods are not attaining a good detector’s performance.

## 1. Introduction

The number of mobile devices is remarkably growing year over year. For instance, the number of mobile devices reached 8.6 billion devices at the end of 2017, up from 7.3 billion devices at the end of 2014 and it is expected to exceed 12.3 billion devices at the end of 2022. Furthermore, the global mobile data traffic was almost 15 exabytes per month at the end of 2018, up from 3.7 exabytes per month at the end of 2015 and it is projected to be 77.5 exabytes at the end of 2022. It is also foreseeable that over 400 million devices are going to be fifth generation (5G) capable and about 12% of a global mobile data will be on the 5G cellular connectivity by 2022 [1,2,3]. 5G networks will attain 1.5 billion subscriptions in 2024 [4]. Massive multiple-input multiple-output (M-MIMO), together with other technologies, is a auspicious technology to meet high data rate, ultra-low latency, broader coverage 5G system requirements [5]. M-MIMO can also reinforce the spectrum and power efficiencies [6,7,8]. It also increases the throughput of wireless networks. For instance, when the number of user terminals is large, channel time spent on channel state information (CSI) feedback can bury the channel time spent of transmission of data. Therefore, a scalable user selection mechanism has been proposed in dense use population to enhance the overall throughput [9]. In addition, distributed MIMO eliminates the interference and improves the throughput in wireless communication networks [10]. The capacity enhancement is also possible by furnishing the base station (BS) with extra antennas [11]. In [12], influence of different number of antennas on the performance is comprehensively illustrated. However, along with attractive advantages of the M-MIMO system, the optimal detection methods such as the maximum likelihood (ML), encounter a high complexity in case of higher constellations (i.e., 64QAM) and higher number of antennas (>16 and more), which prohibits the ML in realization. The literature is rich with M-MIMO detection schemes to balance the performance and the computational complexity. For example, a survey dated 2015 [13] offered a comprehensive illustration of MIMO detection basics and concepts, and illustrated the half-a-century history of detection schemes for MIMO technology. Another comprehensive paper can be found [14] wherein an intensive comparison between linear and non linear methods-based M-MIMO detection have been provided. For instance, a detector based on sphere decoding (SD) can be found in [15,16,17,18]. The fixed-complexity SD would also suffer from high complexity with large number of antenna elements [19]. In [20], dominance conditions are taken into consideration to propose an efficient king SD algorithm where the computational complexity is significantly reduced. In [21], the branch-and-bound algorithm is also developed with the dominance conditions where the channel matrix properties are exploited to reduce the complexity. In [22], SD and single tree-search scheme is proposed where extrinsic log-likelihood ratios (LLRs) are used to achieve convinced balance between the performance and the complexity. Approximate expectation propagation (EP) is proposed in [23]. In [24], a detector based on likelihood ascent search (LAS) was proposed for M-MIMO. Although they achieve a good performance, the complexity is still high. A class of linear detection methods attracted the researchers’ attention because of low complexity. However, linear detectors have a considerable performance loss and high complexity in ill-conditioned environment. They also affords inverse of the matrix which is not a hardware-friendly. In the literature, approximate matrix inversion methods are illustrated to avoid the burden of exact computation of the matrix inversion [25,26,27,28,29]. In [30,31], a discrete sorting optimization scheme with QR decomposition is proposed for UL M-MIMO system where all simulation was conducted in QuaDRiGa.

In this paper, several iterative matrix inversion methods are exploited to detect the signal and is illustrated in real scenarios. QUAsi Deterministic RadIo channel GenerAtor (QuaDRiGa) package [32] is used in the simulation to compare among iterative matrix inversion methods. In realistic scenario, we provide a comparison between the Neumann series (NS), the Gauss-Seidel (GS), the successive overrelaxation (SOR) method, the Jacobi (JA) method, the Richardson (RI) method, the optimized coordinate descent (OCD) method, and the conjugate-gradient (CG) method. In the QuaDRiGA, large n is required to obtain an acceptable performance. This paper also shows that when β≈1, iterative matrix inversion methods are not attaining a good performance, where β is the ratio between the user antennas and BS antennas.

This paper is arranged as: Section 2 illustrates the M-MIMO model, definitions, and fundamentals of linear detectors. Section 3 exhibits the approximate matrix inversion methods. Section 4 shows the complexity analysis of iterative matrix inversion methods. In Section 5, numerical results are presented. Section 6 presents the future trend, research challenges, and concludes the paper. Table 1 illustrates the notations and corresponding full meaning.

## 2. Overview

The fundamental communications theoretic concepts of MIMO detection date back to 1960s, although the term was not used at that time. During the last half a century, significant research efforts were made on MIMO detection by the wireless communication researchers [33]. A detail discussion on the history of MIMO detection is presented in [13]. A plethora of MIMO detector implementation can be found in the literature. The first MIMO detector implementation is presented in [34]. Wong et al. exploited a breadth first *k*-best tree search MIMO detection for a 4×4 MIMO configuration. Garett et al. presented a soft output optimal detector using a parallel architecture [35]. Garett et al. also proposed a depth first sphere decoding (SD) algorithm for 4×4 MIMO systems and 16-QAM [36]. The first minimum-mean square estimation (MMSE) implementation can be credited to Burg et al who proposed an architecture of the 4×4 MMSE in [37]. Burg et al. also proposed the first architecture for the lattice reduction algorithm [38]. The long-term evolution (LTE) specific implementations can be found, e.g., in [39,40]. M-MIMO or large scale MIMO, is an expansion of the ordinary small scale MIMO systems [41,42] where large number of antennas at the BS avails concurrently numerous users with an elasticity to select what users to schedule for reception at any moment. The popular M-MIMO connotation postulates that the user terminals have a solely antenna (the M-MIMO is predominantly inaccurate, because of the single-antenna element. The system is a multiple-input single-output (MISO) downlink or a multiuser single-input multiple-output (SIMO) uplink (UL). As is accustomed in the literature, M-MIMO system will be used in this paper to refer both single and multiple antenna terminal) and that the number of served antennas at the user terminals is remarkably smaller than the number of antennas at the BS. M-MIMO technology is one of the key technologies in 5G and beyond 5G communication systems. M-MIMO system is substantial in implementation of many 5G applications such as the massive machine-type communications (mMTC) where large number of mobile apparatuses is sporadically active [43,44]. In M-MIMO systems, there are an interest in linear detectors because of relative simplicity and low complexity. In this section, the linear detection mechanism is illustrated. It is assumed that the massive MIMO BS antennas *N* is serving *K* single antenna user terminals where N≫K. The channel entries between *N* BS antennas and *K* users forms a channel matrix (**H**) as

(1) $$H=\left[\begin{array}{cccc} h_{11} & h_{12} & \cdots & h_{1j}\\ h_{21} & h_{22} & \cdots & h_{2j}\\ \vdots & \vdots & \vdots & \vdots \\ h_{i1} & h_{i2} & \cdots & h_{ij} \end{array}\right],$$

where $h_{ij}$ presents the channel coefficients (gain\loss) between *ith* receive antenna and *jth* transmit antenna. Each user transmit its symbols individually. The symbol vector $x={\left[x_{1},\;x_{2},\ldots ..,\;x_{K}\right]}^{T}$ presents the transmitted symbols.The corrupted vector $y={\left[y_{1},\;y_{2},\ldots ..,\;y_{N}\right]}^{T}$ is received by the BS receives. This system can be modelled as

(2) y=Hx+n,

where **n** is the additive noise. The column vectors of **H** are assumed to be asymptotically orthogonal. Equation (2) is mostly used in detection approaches, where the channel state information (CSI) is supposed to be perfect at the BS with good synchronization. It is noteworthy that if the instantaneous values of H elements are known from the channel estimation, the detection of x belongs to the family of coherent detection. On the other hand, if the instantaneous channel state estimation is averted, the detection of x is said to be a noncoherent scheme. It should be noted that noncoherent detectors have high computational complexity and an enormous performance loss compared to the coherent detectors because of a degradation in the power efficiency. In M-MIMO detector, the transmitted vector x is retrieved from the received vector y. The ML sequence detection (MLSD) obtains the optimum solution but it exhaustively searches all possible signals as

(3) $$\begin{array}{c} {\hat{x}}_{ML}=\underset{x\in O^{K}}{\arg\;\min}\;\;{∥y-\mathrm{Hx}∥}_{2}^{2}. \end{array}$$

The ML scheme has an exponential computational complexity in the number of decision variables $O^{K}$ and therefore, it is prohibitively complex in massive MIMO.For example, if a transmitter has four antennas and using 64-QAM scheme, it needs a $16.7\times 10^{6}$ comparisons if the ML detection is used. Linear detectors can solve the problem in (3) with convex optimization methods to obtain the quasi-optimal solution. They are relatively simple in implementation, but they afford a considerable performance loss in high loaded systems. Furthermore, if the system size is large, the required matrix inversion becomes complex and approximations may be needed. In linear detectors, received signal y is multiplied with the equalization matrix $A^{H}$, $\hat{x}=S\left(A^{H}y\right)$, followed by a slicer S(.) to quantize every element to the closest neighbour in the constellation [45]. In this section, we present the most popular linear detectors, i.e., the matched filter (MF), the zero-forcing (ZF) and the MMSE.

### 2.1. MF-Based Detector

In MF, the estimated signal is given as

(4) $${\hat{x}}_{MF}=S\left(H^{H}y\right).$$

The MF works truly when *N* is much larger than *K* but it obtains the worst performance compared to other linear detectors. The MF-based detector maximizes the received signal-to-noise ratio (SNR) of each stream by ignoring the impact of interference. In case of ill-conditioned channels, the performance is badly deteriorated for a square M-MIMO system [46].

### 2.2. ZF-Based Detector

In the ZF-based detector, the aim is to make the received signal-to-interference ration (SINR) as large as possible. However, the channel matrix H is inverted and hence, taking off the impact of the channel [47]. The equalization matrix depends on the Moore-Penrose pseudo-inverse ($H^{+}$) and it is given as

(5) $$A_{ZF}^{H}={\left(H^{H}H\right)}^{-1}H^{H}=H^{+}.$$

However, to avoid the square channel matrix (H) scenario, β has to be small. In the ZF-based detector, the signal can be estimated as

(6) $${\hat{x}}_{ZF}=S\left(A_{ZF}^{H}y\right).$$

The ZF detector discards the noise effects and it works fairly in interference-limited scenarios with high computational complexity. In a small-valued coefficient channel, the ZF- and MF-based detectors may have a noise enhancement. Therefore, the minimum mean square error (MMSE)-based detector is proposed to take the noise effect in the equalization process.

### 2.3. MMSE-Based Detector

In an MMSE detector, the mean square error (MSE) between x and $H^{H}y$ is minimized as

(7) $$A_{MMSE}^{H}=\;\arg\;\min_{H\in^{N\times K}}E{∥x-H^{H}y∥}^{2}.$$

The MMSE detector takes into consideration the impact of noise as

(8) $$A_{MMSE}^{H}={\left(H^{H}H+\frac{K}{SNR}I\right)}^{-1}H^{H},$$

where **I** is the identity matrix. In MMSE detector, the signal is estimated as

(9) $${\hat{x}}_{MMSE}=S\left(A_{MMSE}^{H}y\right).$$

The MMSE in (8) relies on a reduction of the noise enhancement and needs an awareness of the SNR [48]. Thus, the MMSE outperforms the ZF- and MF-based detectors. As mentioned earlier, the column vectors of **H** are asymptotically orthogonal, thus, the MMSE detector achieves near-optimal performance.

## 3. Matrix Inversion Methods

A matrix inversion of the *Gram matrix* (G) is mandatory to estimate the signal. However, the computational complexity of linear detectors grows as the size of M-MIMO system rises. In 2013, a new class of detection techniques for M-MIMO is introduced by Wu et al. and approximate matrix inversion method-based UL detector is illustrated in [49]. This detection class has been the most popular of detectors since its initiation in 2013. In M-MIMO, the channel hardening is used to repeal the characteristics of a small scale fading and is being dominant when the number of receive antennas (*N*) is much higher than the number of served users (*K*). For instance, the diagonal entries of $H^{H}H$ grow gradually stronger compered to the non-diagonal entries when the size of the M-MIMO system gets larger [23]. The diagonlisation of the elements in the *Gram matrix*$G=H^{H}H$, where the non-diagonal entries lean to zeros and diagonal components are close to *N* [50,51]. To compute the $G^{-1}$, a complexity of $O\left(N^{3}\right)$ is required, which is not hardware-friendly for M-MIMO. This section presents the concepts of several iterative matrix inversion methods which can be used in low-complexity detectors. It also discusses the pros and cons of each method.

### 3.1. Neumann Series

Neumann series (NS) is a leading solution to approximate the matrix inversion in M-MIMO detector. It takes the benefit of iterative structure to progressively enhance the computing precision of the matrix inversion [52]. The *Gram* matrix $G=H^{H}H$ decomposed into G=D+E, where **D** is the main diagonal entries and **E** is the non-diagonal elements [53,54]. The *Gram* matrix inversion can be approximated as

(10) $$G^{-1}=\sum_{i=0}^{\infty }{\left(-D^{-1}E\right)}^{i}D^{-1},$$

which converges to $G^{-1}$ if $\lim_{i\to \infty }\left(-D^{-1}E\right)=0,$ is fulfilled. In real time applications, a sum of finite terms (*i*) is exploited (10) and hence, fixed number of iterations (n) is required where n is critical in obtaining good accuracy of the matrix inverse which affects the complexity. In [55], channel-aware decision fusion over MIMO channels is illustrated. low complexity sub optimal solution is proposed based on the NS solution. However, it should be noted that the NS method has a abundant performance loss when β≈1. In addition, the convergence of NS method is slow with large number of user terminals. In other words, higher complexity is required which leads to inaccurate matrix inversion and hence, the detector experiences a significant loss.

### 3.2. Gauss-Seidel

The GS or the successive displacement, is an iterative method to avoid the matrix inversion [56]. In each iteration, it uses the most up-to-date estimation from the previous iteration. In the GS detector, the Hermitian positive definite matrix (A) is decomposed into A=D+L+U where **D**, **L** and **U** are the diagonal elements, the strictly lower triangular entries, and the strictly upper triangular entries, respectively. GS iterative method can estimate the signal ($\hat{x}$) as

(11) $${\hat{x}}^{\left(n\right)}={\left(D+L\right)}^{-1}\left({\hat{x}}_{MF}-U{\hat{x}}^{\left(n-1\right)}\right),\;n=1,2,\cdots$$

where ${\hat{x}}_{MF}$ is the output of MF method. To obtain fast convergence rate and hence, reduce the complexity, initialization is mandatory in GS detector. If the initial values ${\hat{x}}^{\left(0\right)}$ are not well known, they can be considered zeros [57]. The GS detector is not desired in parallel implementation because of the internal sequential iterations structure [58]. The GS detector achieves better performance than the NS detector with lower complexity.

### 3.3. Successive Overrelaxation

The GS detector has a performance loss. Therefore, the SOR method is used because of the flexibility to achieve a good performance [59]. The SOR method is also an iterative method to avoid matrix inversion where the signal is estimated as

(12) $$\begin{array}{c} {\hat{x}}^{\left(n\right)}={\left(\frac{1}{\omega }D+L\right)}^{-1}\left({\hat{x}}_{MF}+\left(\left(\frac{1}{\omega }-1\right)D-U\right){\hat{x}}^{\left(n-1\right)}\right), \end{array}$$

where ω is the relaxation parameter and has a considerable impact in obtaining a high performance within a small n. A suitable value of ω is required for convergence. If ω=1, the SOR method will be equivalent to the GS method. In general, the SOR method is convergent when 0<ω<2 [60]. In addition, the GS and SOR methods are not readily implemented on parallel computing platforms because the triangular systems have to be solved at every iteration.

### 3.4. Jacobi Method

In JA method, the signal estimation in a diagonally dominant system is obtained as

(13) $${\hat{x}}^{\left(n\right)}=D^{-1}\left({\hat{x}}_{MF}+\left(D-A\right){\hat{x}}^{\left(n-1\right)}\right),$$

which holds if:

(14) $$\lim_{n\to \infty }{\left(I-D^{-1}A\right)}^{n}=0.$$

The initial estimation (${\hat{x}}^{\left(0\right)}$) can be used as

(15) $${\hat{x}}^{\left(0\right)}=D^{-1}{\hat{x}}_{MF}.$$

The JA detector obtains a good performance when β is small. In general, it can be easily implemented for parallel computation. In numerical methods, it is well known that the convergence speed of the JA method is slower than the convergence speed of the GS and SOR methods. In [61], the convergence speed of the conventional JA method has been improved by a decision-aided JA method.

### 3.5. Conjugate-Gradient Method

The CG is another method to avoid the matrix inversion and is an example par excellence of a Krylov subspace method. The CG detector can estimate the transmitted signal as

(16) $${\hat{x}}^{\left(n+1\right)}={\hat{x}}^{\left(n\right)}+\alpha^{\left(n\right)}p^{\left(n\right)},$$

where $p^{\left(n\right)}$ is the conjugate direction with paying attention to **A**, i.e.,

(17) $${\left(p^{\left(n\right)}\right)}^{H}Ap^{\left(j\right)}=0,\;for\;n\neq j,$$

and

(18) $$p^{\left(n\right)}={\hat{x}}_{MF}^{\left(n\right)}+\frac{{\hat{x}}_{MF}^{\left(n\right)}.{\hat{x}}_{MF}^{\left(n\right)}}{{\hat{x}}_{MF}^{\left(n-1\right)}.{\hat{x}}_{MF}^{\left(n-1\right)}}p^{\left(n-1\right)},$$

and $\alpha^{\left(n\right)}$ is a scalar parameter as

(19) $$\alpha^{\left(n\right)}=\frac{{\hat{x}}_{MF}^{\left(n\right)}.{\hat{x}}_{MF}^{\left(n\right)}}{A{\hat{x}}_{MF}^{\left(n-1\right)}.{\hat{x}}_{MF}^{\left(n-1\right)}}.$$

The CG method is numerically robust and can operate much better under close to ill-conditioned channel than the other algorithms [62]. It usually performs better than the NS and JA methods. It is also implemented in Xilinx Virtex-7 FPGA for a 128×8 in [63]. However, it suffers from low parallelism and considerable correlation issues [64].

### 3.6. Richardson Method

In the RI method, symmetric matrices are used and defined as positive at their execution. Similar to the SOR method, it is overly sensitive to a relaxation parameter (ω) to achieve faster convergence where $0<\omega \leq \frac{2}{\lambda }$ and λ is the largest eigenvalue of **H** [64]. The signal is estimated as

(20) $$x^{\left(n+1\right)}=x^{\left(n\right)}+\omega \left(y-{\mathrm{Hx}}^{\left(n\right)}\right)\;n\;=\;0,\;1,2,\;\cdots .$$

In a Richardson-based detector, the initial solution $x^{\left(0\right)}$ can be set as a zero vector without loss of generality as no prior knowledge of the final solution is available [65]. A constant-valued relaxation parameter (ω) has high impact to achieve a satisfactory performance [66,67]. The value of ω can be determined by the eigenvalues. A detector based on the RI method is a hardware-friendly and decreases the complexity from $O\left(K^{3}\right)$ to $O\left(K^{2}\right)$ [68]. However, a satisfactory performance can be achieved when n is large which increases the complexity as well.

### 3.7. Optimized Coordinate Descent Method

Coordinate descent (CD) attains an approximate solution of large number of convex optimization using series of coordinate-wise updates. It employs the single-variable to refine the estimated signals sequentially where the estimated signal is given as

(21) $${\hat{x}}_{k}={\left({∥h_{k}∥}^{2}+N_{0}\right)}^{-1}h_{k}^{H}\left(y-\sum_{j\neq k}h_{j}x_{j}\right),$$

A pre-processing and refinements are offered to reduce the operations within each iteration and this is called optimized CD (OCD). A low complexity detector based on the OCD method is implemented in a high-throughput FPGA design for M-MIMO systems with high use efficiency [69,70].

## 4. Complexity Analysis

In complexity analysis, the most dominant mathematical operations are the number of divisions and number of multiplications. To compute the $D^{-1}$, *K* real number of divisions are required. The computational complexity of the NS method is $O\left(K^{3}\right)$ while the RI, the SOR, the GS, the JA, and the CG methods require $O\left(K^{2}\right)$. The OCD-based detector requires the lowest complexity of $O\left(K\right)$. Table 2 compares between the complexity of detectors based on several approximate matrix inversion methods.

## 5. Results and Discussion

The performance and the complexity of the NS, GS, SOR, JA, RI, OCD, and CG detectors will be described. A comparison between the iterative matrix inversion methods-based M-MIMO detector will be provided in bit-error-rate (BER) performance, the SNR, and the number of multiplications. In all simulations, we consider urban macro-cell line of sight (LOS) channels generated by QuaDRiGA to generate realistic radio channel impulse responses for system-level simulations of mobile radio networks. Depending on the angular spread and the amount of diffuse scattering, the typical value of clusters is around 10 clusters for the line-of-sight (LOS) propagation environment and 20 clusters for non-LOS. The angular spread values around 20–90 degrees and the carrier frequency is 2 GHz. The configuration of M-MIMO systems with user terminals and BS antennas are 16×128, 32×128, and 64×128 and the modulation scheme is 64QAM.

**Figure 1:**
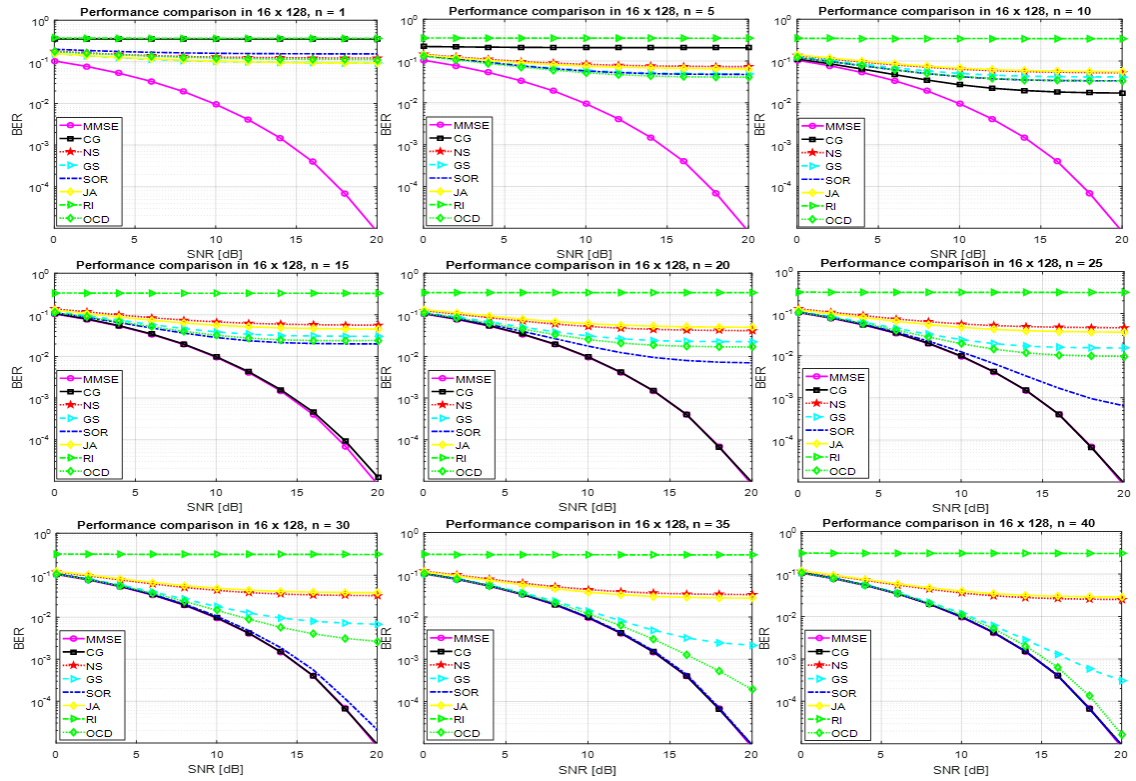
Performance of a detector based on several approximate matrix detection methods and the exact MMSE method for 16×128 M-MIMO system and 64QAM.

Figure 1 shows the performance of a detector based on several approximate matrix inversion methods at 16×128 MIMO where $\beta =\frac{16}{128}=0.125\ll 1$). In such scenario, a detector based on approximate matrix inversion methods needs high n (i.e., n>10) to achieve a satisfactory performance. At n=15, the detector based on the CG method achieved a good performance while other methods required higher n to achieve an acceptable performance. The CG method is numerically robust even when the channel is ill-conditioned. The SOR and OCD methods are obtained BER = $10^{-2}$ at SNR = 15 dB at n=20 and n=25, respectively. It is noteworthy that the NS method, RI method and JA method are not attaining a satisfactory performance in realistic radio channels.

**Figure 2:**
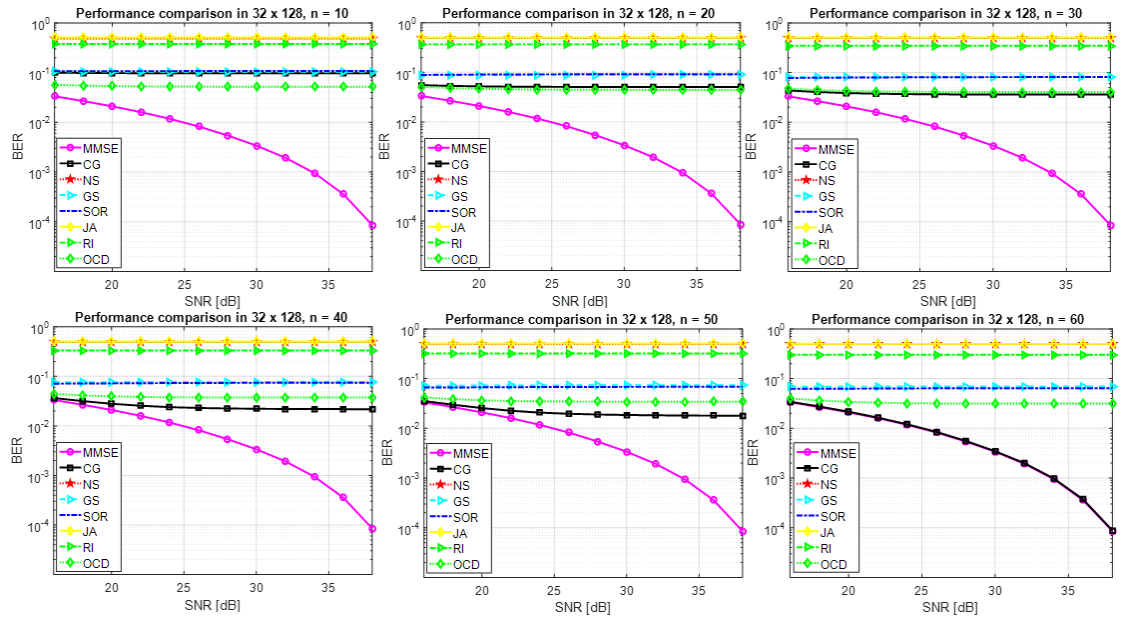
Performance of a detector based on several approximate matrix detection methods and the exact MMSE method for 32×128 M-MIMO system and 64QAM.

Figure 2 presents the performance profile when $\beta =\frac{32}{128}=0.25$. In this case, unsatisfactory performance is obtained. However, the CG method achieves the MMSE performance at high n (i.e.,n=60). A detector based on other methods does not obtain a satisfactory performance even in case of high n.

**Figure 3:**
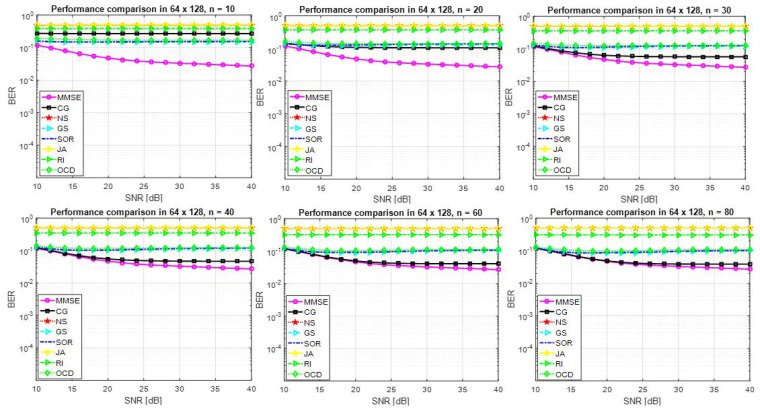
Performance of a detector based on several approximate matrix inversion methods and the exact MMSE method for 64×128 M-MIMO system and 64QAM.

Figure 3 shows that the performance of several approximate matrix inversion methods when $\beta =\frac{64}{128}=0.5$. It is clear that the performance of the detector is not satisfactory and the approximate matrix inversion methods are not numerically robust when the number of users is relatively high compared to the number of antennas at the BS. However, high n is required to achieve BER = $10^{-1}$.

**Figure 4:**
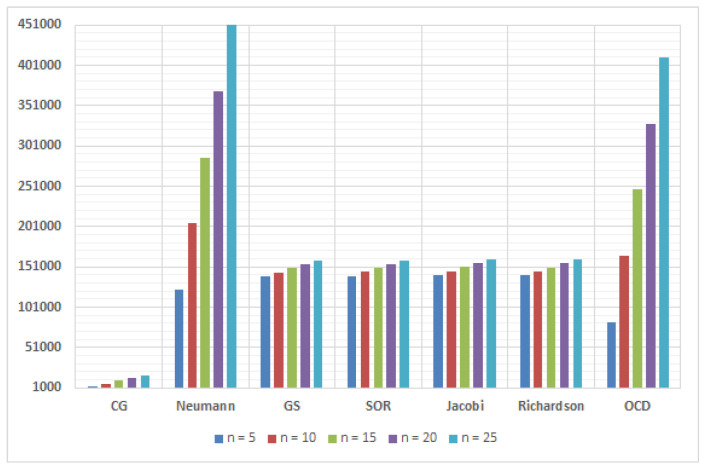
Number of multiplications among several approximate matrix inversion methods in 16×128 MIMO.

Figure 4 presents the number of multiplications among the approximate matrix inversion methods. It is clear that the CG method has the lowest number of multiplications and it has the best performance as mentioned in Figure 1. On the other hand, the NS method and the OCD method had the highest number of multiplications and they need more iterations to converge.

## 6. Conclusions and Future Directions

This research could be extended by investigating different M-MIMO setup. For instance, different number of LOS and non-LOS clusters, different angular spread values, and different carrier frequencies could be considered.

To improve the performance-complexity trade-off, a deep learning (DL)-based sphere decoding (SD) for M-MIMO UL data detection has to be studied where the radius of the hypersphere could be intelligently learnt by a deep neural network (DNN). In addition, use of approximate matrix inversion methods, such as the NS and Newton iteration (NI) methods, should be investigated to reduce the computational complexity by reducing the searched space which make the SD algorithm more efficient. We expect that use of the DNN and approximate matrix inversion methods will achieve a quasi-optimal performance with low computational complexity. Machine learning can be used to select the best algorithm to be applied rather than finding the best signal estimation. In addition, the performance of a sparsity-based M-MIMO detection has to be investigated with the iterative matrix inversion methods.

This paper studied a detector based on several iterative matrix inversion methods in realistic radio channel, QuaDRiGA. It is illustrated that such methods required high n to achieve a satisfactory performance when β≪1. The CG method achieves better performance over other methods and is robust in realistic radio channels, while NS method, RI method and JA method did not achieve satisfactory performance. However, such methods are not numerically robust and did not achieve a satisfactory performance when the number of users is relatively high compared to the number of antennas at the base station. In complexity analysis, the CG method achieves lowest number of multiplications and has the best performance, while NS method and the OCD method have the highest number of multiplication.

## Figures and Tables

**Table 1:** Notation and corresponding full meaning.

Notation	Meaning
β	ratio between user antennas and BS antennas
5G	fifth generation
*K*	number of user terminals
*N*	number of BS antennas
x	transmitted symbol vector
y	received symbol vector
S(.)	slicer
**n**	additive white Gaussian noise (AWGN)
**H**	channel matrix
$O^{K}$	decision variables
**A**	equalization matrix
$H^{+}$	Moore-Penrose pseudo-inverse
**G**	Gram matrix
**D**	Diagonal matrix
**E**	non-diagonal matrix
**L**	lower triangular matrix
**U**	upper triangular matrix
ω	relaxation parameter
n	number of iterations

**Table 2:** Complexity comparison among approximate matrix inversion methods.

Method	Number of Multiplications
NS	$4K^{3}\left(n-2\right)+\left(2K+1\right)K^{2}+\left(4N-1\right)K$
RI	$\left(4N+4n\right)K^{2}+2KN$
SOR	$\left(4N+4n-2\right)K^{2}+2\left(N-n+1\right)K$
GS	$\left(4N+4n-2\right)K^{2}+2\left(N-2n+1\right)K$
OCD	(8NK+4K)n
JA	$\left(4N+4n+1\right)K^{2}$+2NK
CG	$(N+2K^{2})n$
